# Comment on “Relative Diffusivities of Bound and Unbound Protein Can Control Chemotactic Directionality”

**DOI:** 10.1021/acs.langmuir.1c02840

**Published:** 2022-02-17

**Authors:** Jaime Agudo-Canalejo, Pierre Illien, Ramin Golestanian

**Affiliations:** † Department of Living Matter Physics, Max Planck Institute for Dynamics and Self-Organization, D-37077 Göttingen, Germany; ‡ Sorbonne Université, CNRS, Laboratoire Physicochimie des Electrolytes et Nanosystèmes Interfaciaux (PHENIX), UMR 8234, 4 place Jussieu, 75005 Paris, France; § Rudolf Peierls Centre for Theoretical Physics, University of Oxford, Oxford OX1 3PU, United Kingdom

In a recent study^1^ published in *Langmuir*, Mandal and Sen claim to propose a “new” kinetic model to analyze the directional movement of enzyme molecules in response to a gradient of their substrate, with the supposedly new prediction that net movement occurs up the substrate gradient when the diffusivity of the substrate-bound enzyme is lower than that of the unbound enzyme and movement occurs down the substrate gradient when the diffusivity of the substrate-bound enzyme is higher than that of the unbound enzyme. In this Comment, we point out that the same result and prediction (with an identical derivation) were already obtained by us as one of the central results in ref (2), whose abstract indeed states that we found “a new type of [chemotactic] mechanism due to binding-induced changes in the diffusion coefficient of the enzyme” which “points toward lower substrate concentration if the substrate enhances enzyme diffusion and toward higher substrate concentration if the substrate inhibits enzyme diffusion.”

This would not require any additional explanation had Mandal and Sen been unaware of our work because the rediscovery of known phenomena is a common-enough occurrence in science. However, Mandal and Sen repeatedly cite and discuss ref (2), widely misrepresenting it and falsely claiming (in order of appearance) that our approach

• “[assumes] that the effective diffusivity of the protein is the weighted average of the diffusivity of free and bound protein”;
• “[does not make] a distinction between the mass fluxes of the free and the bound protein”;
• “is in contrast with [their approach]”;
• “fails to recognize the gradients of the free and bound protein that are created because of the presence of the ligand gradient”;
• “seriously underestimates the chemotaxis of the protein when there is no initial gradient of the protein in the system”; and
• “[ignores] two terms that are incorporated in [their eq 6]”

As we show below, the derivation and, consequently, the central result (eq 6) of Mandal and Sen are *identical* to those in ref (2); therefore, all of their claims listed above are unjustified.

We begin by noting that our derivation in ref (2) starts from a fully stochastic description of the enzyme and substrate molecules and furthermore includes the possibility of hydrodynamic and nonspecific enzyme–substrate interactions. After making a mean field approximation for the substrate concentration, it is shown that the combination of nonspecific and hydrodynamic interactions results in an additional *phoretic* mechanism for chemotaxis that is not taken into account by Mandal and Sen. The results of Mandal and Sen are therefore a special case of our results (corresponding to setting ***v***_e_ = ***v***_c_ = 0 in eqs 6, 7, and 15 of ref (2)). In what follows, we discuss only this special case.

The equivalence in notation between our work^2^ and Mandal and Sen’s^1^ is summarized in Table 1, and the equivalence between equations, which for the purpose of this Comment we will number (I–IV), is summarized in Table 2. By simply contrasting the versions of (I), (II), and (III) in ref (1) with those of ref (2), it is obvious that they are manifestly identical. Because (IV), which is the central result in both works, is directly derived from (I–III) in exactly the same way in both works, it must necessarily be identical in both works as well. Any illusory perception of Mandal and Sen’s results being different from ours must thus come from the way that (IV) is presented in each case.

**Table 1:** Equivalence Table for Notation

meaning	ref ^1^	ref ^2^
free enzyme concentration	*c*_A_	ρ_e_
enzyme–substrate complex concentration	*c*_AB_	ρ_c_
total enzyme concentration	*c*_A_^T^ = *c*_A_ + *c*_AB_	ρ_e_^tot^ = ρ_e_ + ρ_c_
substrate concentration	*c*_B_	ρ_s_
free enzyme diffusion coefficient	*D*_A_	*D*_e_
enzyme–substrate complex diffusion coefficient	*D*_AB_	*D*_c_
substrate binding rate	*k*_1_	*k*_on_
substrate unbinding rate	*k*_–1_	*k*_off_
dissociation constant	*K*_d_ = *k*_–1_/*k*_1_	*K* = *k*_off_/*k*_on_

**Table 2:** Equivalence Table for Equations

	meaning	ref ^1^	ref ^2^
(I)	evolution of free enzyme concentration	eq 2	eq 6
(II)	evolution of enzyme–substrate complex concentration	eq 3	eq 7
(III)	assumption of instantaneous local binding equilibrium	eq 5	eq 11
(IV)	evolution of total enzyme concentration	eq 6	eqs 13–16

In ref (2), we presented (IV) as
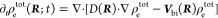
1with the definition of an effective, substrate-concentration-dependent diffusion coefficient
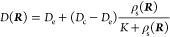
2and a binding-induced chemotactic velocity
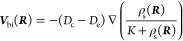
3Equation 1 here has the advantage of being written in a canonical form, with the total enzyme flux being cleanly split into a Fickian diffusion flux  and an advective, chemotactic flux . In particular, in the absence of substrate gradients, the latter chemotactic flux vanishes and one is left with Fickian diffusion only.

The result for (IV) of Mandal and Sen^1^ is identical to this one but is just presented in a noncanonical form that mixes diffusive and chemotactic fluxes. Indeed, by inserting the expressions for *D*(***R***) and ***V***_bi_(***R***) into eq 1 and rearranging the gradient terms, one can trivially rewrite eq 1 as
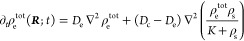
4which now makes it explicit that Mandal and Sen’s result is identical to ours. This form of the equation is not particularly transparent, however, because the second term also contributes to diffusion and is nonzero even if the substrate concentration is uniform in space.

For completeness, we note that there are other instructive ways in which this same evolution equation can be written. For example, in ref (3), we pointed out that it can also be equivalently rewritten as
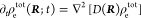
5with *D*(***R***) given by eq 2, which implies that, in the absence of enzyme sources and sinks and in the presence of an externally maintained substrate gradient, the enzyme concentration will reach a zero-flux stationary state with , i.e., it will accumulate in regions where the effective diffusion coefficient is lowest.

In summary, Mandal and Sen^1^ seem to have misunderstood the results in ref (2), which are identical to theirs (although ref (2) additionally includes the possibility of phoresis arising from nonspecific and hydrodynamic interactions). Although in light of this the central message of Mandal and Sen (i.e., that “relative diffusivities of bound and unbound protein can control chemotactic directionality” as per the title) is not new, we note that their work does bring some new and interesting aspects to the literature, in particular, (i) the inclusion of the catalytic step (with catalytic rate *k*_2_ in ref (1) and *k*_cat_ in ref (2)) which was neglected in ref (2) (by considering the limit *k*_cat_ ≪ *k*_off_) and (ii) their numerical simulation of the transient kinetics in a setting that mimics a microfluidics experiment, which moreover helps to ascertain the range of validity of the instantaneous local binding equilibrium assumption.

To finish, we note that since the publication of ref (2) there have been some further developments of the idea of chemotaxis resulting from binding-induced changes in diffusivity. In refs (4) and (5), it was shown that the same mechanism operates for nonrigid enzymes or proteins that undergo shape fluctuations, in which case the binding-induced changes in diffusion that cause chemotaxis can come not only from changes in the average shape of the protein but also from changes in the magnitude of its shape fluctuations. In ref (5), it was explicitly shown that the competition between phoretic and binding-induced mechanisms for chemotaxis can lead to the accumulation or depletion of enzymes not just in regions of highest or lowest substrate concentration but also in regions with an intermediate, tunable critical substrate concentration. Finally, in ref (6) it was shown that a similar mechanism for chemotaxis due to changes in diffusivity operates in the case of oligomeric proteins that can reversibly associate and dissociate into monomers. Such oligomeric proteins spontaneously accumulate in regions in which the oligomeric (slowly diffusing) form is most stable, a process called “stabilitaxis”.
